# Retraction: Natural Cross Chlamydial Infection between Livestock and Free-Living Bird Species

**DOI:** 10.1371/annotation/9b33ec1f-9579-41c0-bede-3a38ee6af21c

**Published:** 2012-10-08

**Authors:** Jesús A. Lemus, Juan A. Fargallo, Pablo Vergara, Deseada Parejo, Eva Banda

Juan A. Fargallo, Pablo Vergara, Deseada Parejo and Eva Banda, as co-authors of the article published in PLOS ONE (2010) titled “Natural Cross Chlamydial Infection between Livestock and Free-Living Bird Species”, request the retraction of this publication.

The Ethics Committee of the Spanish Superior Council of Scientific Research (CSIC) has carried out an investigation in relation to concerns about potential scientific misconduct by Jesús A. Lemus, who was also a co-author of this article. The investigation has questioned the validity of the laboratory analyses conducted by Dr. Lemus in relation to this study, and was unable to establish at which laboratories the analyses were conducted. 

Specifically, the authors have been unable to verify the validity of the Chlamydophila analyses for sheep abortions, sheep faeces, sheep stable dust, kestrel nest dust and insects. There are also concerns about the validity of the results obtained for the Chlamydophila serovar characterization, genetic diversity and MSLT analyses for Chamydophila. As a result, the authors wish to retract the article.

